# On the quality of quantitative instruments to measure digital competence in higher education: A systematic mapping study

**DOI:** 10.1371/journal.pone.0257344

**Published:** 2021-09-10

**Authors:** Rafael Saltos-Rivas, Pavel Novoa-Hernández, Rocío Serrano Rodríguez

**Affiliations:** 1 Facultad de Filosofía Letras y Ciencias de la Educación de la Universidad Técnica de Manabí, Portoviejo, Ecuador; 2 Escuela de Ciencias Empresariales, Universidad Católica del Norte, Coquimbo, Chile; 3 Facultad de Ciencias de la Educación, Universidad de Córdoba, Cordoba, Spain; University of Granada: Universidad de Granada, SPAIN

## Abstract

In this study, we report on a Systematic Mapping Study (SMS) on how the quality of the quantitative instruments used to measure digital competencies in higher education is assured. 73 primary studies were selected from the published literature in the last 10 years in order to 1) characterize the literature, 2) evaluate the reporting practice of quality assessments, and 3) analyze which variables explain such reporting practices. The results indicate that most of the studies focused on medium to large samples of European university students, who attended social science programs. Ad hoc, self-reported questionnaires measuring various digital competence areas were the most commonly used method for data collection. The studies were mostly published in low tier journals. 36% of the studies did not report any quality assessment, while less than 50% covered both groups of reliability and validity assessments at the same time. In general, the studies had a moderate to high depth of evidence on the assessments performed. We found that studies in which several areas of digital competence were measured were more likely to report quality assessments. In addition, we estimate that the probability of finding studies with acceptable or good reporting practices increases over time.

## 1 Introduction

In a world governed by Information and Communication Technologies (ICTs) [1], most of the essential processes of modern society are automatized in one way or another [2]. For this reason, it is essential that professionals have sufficient ICT skills and competencies, adapting to the demands of the modern working world [3, 4]. Higher education institutions play an essential role in this context [5], that is, by integrating different strategies to provide these digital competencies to their educational community [6] and building coherent evaluation processes and instruments. The latter is particularly important not only for diagnosing the educational community, but also to verify the extent to which an intervention program has been effective.

The act of evaluating digital competencies is, from a theoretical perspective, a measurement task. As a consequence, it largely depends on the quality of the employed instrument. Regardless of the structure of the instrument, its quality is given by the fulfillment degree of two psychometric properties: reliability and validity [7]. As recent reviews showed [8, 9], the topic of digital competence in higher education comprises of a large and fertile body of studies. This literature has been characterized on several occasions from different perspectives: ranging from concept use [10, 11] to organizational infrastructures, strategic leadership, and teaching practices [12].

However, the evaluation process itself and, more specifically, the quality of the employed instrument are two important topics that have not been extensively addressed in the past. So far, there is still an uncertainty about how and to what extent studies ensure that the instruments used are adequate to measure digital competencies in higher education. In our opinion, characterizing current literature on these issues is relevant for both researchers and practitioners. In the first case, researchers are provided with an overview on 1) the main studies’ features and 2) the trends in reporting quality assessments. As a consequence, current literature is assessed and some important research opportunities to explore in the near future are identified. Additionally, such characterization would help us to reflect on what we have done well and what we have not done when conducting or reporting on quality assessments. In the case of practitioners, since literature is assessed according to how the quality of the employed instruments has been assured, they are provided with a top-quality list of studies that serve as a good starting point for reusing previous experiences.

In order to shed light on these issues, in this paper, we conducted a systematic mapping study [13]. More specifically, we aim at 1) characterizing the literature demographically and methodologically, 2) describing how and to what extent studies assured the quality of employed instruments, and 3) identifying what studies’ features explain certain reporting practices. We specifically focused on literature using quantitative instruments in the form of questionnaires. The rationale behind this move, as noted by [11], is that these data collection methods are among the most commonly employed by researchers in this field. Therefore, it is expected that the results obtained can characterize the vast majority of published studies on this topic.

## 2 Digital competencies in higher education

Currently, no consensus exists about what entails digital competencies in the context of higher education [9–11]. This is mainly because, as noted by [11], the definition of digital competence is context-dependent and, therefore, it is possible to find various positions both in the scientific context [14] and in the of the definition of government policies [3]. In addition to this, we have to take into account that students and academic staffs demand for specific competencies, which are not necessarily the same [8].

Among the thirty definitions reviewed in [11], perhaps the most complete is the one provided by [3] in the context of defining policies. The author defined digital competencies as follows: “the set of knowledge, skills, attitudes, strategies and awareness which are required when ICT and digital media are used to perform tasks, resolve problems, communicate, manage information, collaborate, create and share content, and build knowledge in an effective, efficient and adequate way, in a critical, creative, autonomous, flexible, ethical and a sensible form for work, entertainment, participation, learning, socialization, consumption and empowerment.” (p. 3).

In the same line, but in a scientific context [10], defined it as the composition of the following: “(1) technical competence, (2) the ability to use digital technologies in a meaningful way for working, studying and in everyday life, (3) the ability to evaluate digital technologies critically, and (4) motivation to participate and commit in the digital culture.” (p. 655).

Another definition of digital competence can be realized by approaching this concept through the frameworks defined in both the scientific context [8] and in that of government policies [15]. A notable example in the latter case is the *Digital Competence Framework for Citizens* (DigComp) created by the European Commission. This framework has gone through 3 fundamental versions. In the first one, DigComp 1.0 [16], 21 digital competencies (dimension 2) described in terms of knowledge, skills and attitudes were considered (dimension 4). Moreover, these competencies were organized in 5 areas (dimension 1): Information, Communication, Content-creation, Safety and Problem-solving. To assess how competent a citizen is, DigComp 1.0 proposes three proficiency levels (dimension 3): A (foundation level), B (intermediate level) and C (advanced level). The framework also includes examples of use on the application of digital competencies for different purposes (dimension 5).

The main contribution of the second version, DigComp 2.0 [17], was the redefinition of some concepts and terms (dimensions 1 and 2). However, it maintained the five areas of digital competencies of the first version. Finally, the third version, which was named DigComp 2.1 [18], established 8 levels of proficiency (dimension 3) instead of the 3 defined by DigComp 1.0. These levels were defined with consecutive numbers from 1 to 8 with the following distribution: Foundation (1 and 2), Intermediate (3 and 4), Advanced (5 and 6) and Highly specialized (7 and 8). It is important to note that DigComp 2.1 did not include an update to dimension 4 related to knowledge, skills and attitudes. Instead, the authors focused more on dimension 5, that is, by showing examples of use of the framework in the employment and learning contexts.

From the above-mentioned definitions, it is easy to conclude that measuring the degree or level of digital competence involves at least three areas: knowledge, skills and attitudes. In other words, the development of an instrument for measuring digital competencies should include assessment items related to these three competence areas.

Existing literature on the subject includes several works related to our research topic and type (secondary study). Table 1 summarizes the current related studies. These studies were selected from the systematic search explained in Sec. 4, but considering only secondary studies published in the period 2016-2020.

**Table 1:** Review of articles published in the last 5 years on digital competence 1) with a systematic review (SR), 2) covering higher education (HE), and 3) addressing digital competence evaluation (DCE).

Study	Research topic	Period covered	Studies included	SR	HE	DCE
[19]	Digital competence in Latin America.	2012-2017	11	Yes	No	Yes
[8]	Digital competence in Spanish teachers.	NS(*)	NS	No	No	Yes
[11]	Concept use in higher education.	1997-2017	107	Yes	Yes	Partial
[22]	Students’ information skills.	2014-2018	NS	No	Partial	No
[12]	Policy, organizational infrastructures, strategic leadership, and teaching practices.	2007-2017	41	Yes	Partial	No
[23]	Concept use in higher education from south-western Europe.	2006-2018	41	Yes	Yes	No
[20]	Teaching and learning strategies in higher education.	2014-2017	13	Yes	Yes	Partial
[24]	Digital literacy in teacher education.	2010-2018	37	Yes	Yes	No
[21]	Prevalence of digital competents in higher education from Latin America.	2014-2019	16	Yes	Yes	Partial
[9]	Concept use in higher education.	2009-2018	68	Yes	Yes	No
[25]	Use of ICT in education under learning difficulties.	1975-2019	671	No	Partial	No
[26]	Digital competence in university teachers.	NS	NS	No	Yes	No
[27]	University students’ digital abilities.	2006-2017	126	Yes	Yes	No

* Not explicitly specified in the study.

As seen in the table, only five studies addressed the topic of digital competencies evaluation. They are [8, 11, 19–21]. However [19], did not focus on higher education and the analyzed studies are only from Latin America. Similarly [8], did not focus on higher education and is not a systematic review. In the case of [11], the authors included some relevant factors for the evaluation process, such as the method of data collection (instrument) and the study area of the participants. However, it is not clear which works actually evaluated digital competencies. Regarding these two factors, the authors concluded that most of the studies use mixed methods or surveys for measuring, and are based on populations from different knowledge areas. An important limitation of this study is that it dates back to 2018, so more recent contributions are not present in the review. The study conducted by [20] found that programs and actions developed by the HEIs leave out the development of competencies in content creation and safety. To identify this gap, the authors used the *DigComp 2.1* framework [18] as a reference. Finally, in the meta-analysis developed by [21], the authors found that, in the field of higher education in Latin America, the proportion of students and teachers with digital skills is moderate (64%), with no notable differences between both types of populations.

Regardless of the progress achieved by the above mentioned studies, some important aspects of the process of evaluating digital competencies in higher education remain unexplored. This is the case for the quality of the instruments employed for conducting such an evaluation.

## 3 Quality of quantitative instruments

When developing a quantitative instrument, it is important to assess its quality [7, 28, 29]. This is a process that mainly depends on assessing its reliability and validity [29]. From a psychometric perspective, the first property states whether the instrument provides the same (or similar) results under similar conditions or inputs, while the second one states whether it measures what is supposed to be measured [28]. Several methods to conduct these assessments have been proposed in the past [7] and have been classified into different categories. For instance, the detailed review provided by [30] identified four types of both reliability and validity assessments as shown in Table 2.

**Table 2:** Reliability and validity assessments for quantitative instruments.

Group of assessment	Assessment type	Definition	Typical methods
Reliability	Stability	The extent to which the same results are obtained upon repeated administration of the instrument.	Test-retest reliability
	Internal consistency	How well the different items measure the same characteristic.	Split-half technique, Cronbach’s alpha, Kuder-Richardson formula
	Equivalence	The extent to which parallel administration of the same scale shows consistent results.	The use of the scale by the same administrators at the same time (i.e., inter-rater reliability), administering two parallel forms of the same scales to the same sample successively (i.e., alternative form reliability)
	Scalability	The extent to which individual items in the scale measure the latent trait that is being measured and do so distinctly from other items in the scale.	Mokken scaling
Validity	Face validity	The extent to which the scale is understandable and perceived as relevant by the subjects to ensure their cooperation and motivation.	Not tested using statistical procedures. Subjects, experts or the researcher may be involved in the consideration of whether a scale appears to be relevant
	Content validity	The extent to which the scale adequately samples all possible questions that exist.	Critical review by an expert panel for clarity and completeness or comparison with the literature, or both
	Criterion validity	The extent to which the scale aligns to criterion measures that have been established as valid.	Concurrent validity (information about the criterion that is available at the time the test is administered), predictive validity (information about the criterion measure is obtained after the test has been administered)
	Construct validity	The extent to which the scale correlates with the construct under investigation.	Convergent validity (which uses correlation evidence), factorial/discriminant validity, or discriminant evidence.

Ideally, a study developing or administering an instrument should present enough details about these eight assessments types. However, this is not always possible because the presence of research limitations (e.g., time, lack of another instrument to compare with, access to the participants). In any case, it is important to conduct an assessment of at least one of these types in order to guarantee a suitable degree of consistency and accuracy for the instrument [28]. Even if the instrument have been proposed and validated in a previous study, it is a good practice to check its reliability and validity [28].

### 3.1 Related work on quality evaluation of quantitative instruments

Critically evaluating the quality of quantitative instruments is not a new research topic and has been developed for quite some time in various areas of knowledge, such as education, psychology and health. In what follows, we will review some of the most important reported experiences, emphasizing the conclusions related to the quality of the instruments considered.

In [31] the authors focused on evaluating the quality of the methods used in high-quality trials of continuing medical education. Of the 136 studies selected, only 34.6% reported reliability or validity assessments. In the same context of medical education, in [32] the authors reviewed the instruments and questionnaires used for peer review published up to May 2010. In line with the results of the previous study [31], it was found that most of the questionnaires did not provide sufficient psychometric data.

Smokeless tobacco dependence measures were the focus of the review conducted in [33]. From the 4 selected studies, the authors conclude that the instruments analyzed have limitations in terms of reliability and validity. The same difficulties were detected in the critical synthesis developed in [34] on the so-called Implicit Relational Assessment Procedure, a computer-based psychological measure. From 31 studies published before March 2013, the authors conclude that although there is growing evidence of validity in the studies, they lack sufficient reliability to ensure replicability.

In a more extensive work where 53 studies published in the period 1995-2012 were included [35], reviewed studies that administered the Parenting Style and Dimensions Questionnaire instrument. In this case, the authors highlight that only a few studies involved complex reliability and validity assessments.

The apathy scales validated in generic and specific neurodegenerative disease populations was the focus of the review conducted in [36]. Of the 16 studies analyzed, the authors found a great heterogeneity of results. More specifically, the methodological quality of the studies ranged from poor to excellent.

In an educational context [37], analyzed the validity and reliability of the structured objective clinical evaluation (OSCE) with nursing students. By reviewing 19 papers published up to April 2016, the authors concluded that validity and reliability was adequate in most studies. However, considering that one of the selection criteria in the search conducted by the authors was precisely to include psychometric assessments, this result was somewhat expected. A more objective conclusion is obtained if the 14 studies excluded by the authors that did not meet this criterion are taken into account. In this sense, the 19 studies represent approximately 58% of the relevant studies.

A great heterogeneity of results was also observed by [38] in their evaluation on the replicability, comparability and validity of quality assessment tools for urban green spaces. This work was based on 15 primary studies published up to July 2019.

In [39] the authors summarized the instruments used to measure constructs of marital quality by analyzing 91 primary studies. As the authors indicate, most of the instruments reported include sufficient exploratory evidence of construct validity, but without explicitly defining the construct under study.

In the context of nursing education, the review developed by [40] aimed to determine how valid and reliable simulated patient scenarios are. Relying on 17 studies found in the Cumulative Index to Nursing and Allied Health Literature, the authors conclude that academics are inconsistent in developing both reliable and valid simulated scenarios.

Similarly, in [41] a comprehensive review of the literature was conducted on instruments measuring the competencies of special educators. In total, 20 instruments reported by 29 studies were characterized. The authors found that only 11 instruments (e.g. 55%) have evidence of reliability and validity assessment.

What these experiences tell us is that there are serious problems related to the quality of quantitative instruments. This is an issue that affects several areas of knowledge, including education sciences. However, the current state of the instruments used to measure digital competencies in higher education remains to be known. Thus, the results of our work will allow us to verify, among other things, to what extent this field would be affected by this issue.

## 4 Methodology

This paper follows the methodology described by [13] for conducting systematic mapping studies. In turn, this is a type of secondary study [42]. It is also a correlation study since we aim at analyzing the association between the variables under study.

According to the selected guide, mapping is achieved through three main steps: planning, conducting, and reporting. The following sections describe how the first two steps were developed, while the third one is fulfilled by writing this paper.

The selection and data extraction processes were carried out in parallel by two authors. In order to evaluate the concordance of the results of these processes, we relied on Cohen’s kappa (*κ*). However, it is important to clarify that although we could have used other more sophisticated indicators [43], we consider Cohen’s kappa to be sufficient for our purposes. Our decision is in line with previous research such as [44], where Cohen’s kappa was employed for similar aims.

### 4.1 Research questions

The main goal of this research is to provide an overview of how literature related to the evaluation of digital competencies in higher education reports on the quality of the employed instruments (e.g. questionnaires). We consider the literature published during the period from January, 2010 to July, 2020, which is a time frame commonly used in literature reviews in the field [9, 12, 45].

More specifically, we were interested in answering the following research questions:

- *RQ1)*. What are the main demographic and methodological features of the studies evaluating digital competencies in higher education?
- *RQ2)*. How are quality assessments reported?
  - *RQ2.1)*. What types of assessments and what specific methods are most often reported?
  - *RQ2.2)*. How comprehensive and how deep are these quality reports?
  - *RQ2.3)*. What studies achieve the best balance between coverage and depth?
- *RQ3)*. What studies’ characteristics are more likely associated with certain reporting practices?
  - *RQ3.1)*. How do quality reporting practices evolve over time?

### 4.2 Search

To design the search formula for finding the relevant studies, we proceeded as follows. First, we used the PICO tool [46] to identify the relevant terms according to the population under study, intervention method, comparison group, and outcomes. Second, careful readings of similar reviews such as that of [11, 21] were useful in order to complement the results of applying the PICO tool. As a result, the following search formula was defined:

*(“digital competence” OR “digital literacy” OR “digital literacies”) AND (undergraduate OR postgraduate OR freshmen OR sophomore OR junior OR senior OR preservice OR teacher OR junior OR university OR “higher education” OR college OR tertiary OR “academic staff” OR professor OR lecturer) AND (evaluate OR assess OR appraise OR validate OR evaluation OR assessment OR appraisal OR validation OR evaluation OR assessing OR appraising OR validating)*

This formula was used to search three relevant databases: Scopus, Web of Science, and ERIC (Education Resources Information Center). These databases cover a great part of the scientific literature about Education Sciences and are widely used in the review studies related to this field [11, 12].

The results obtained from applying the above formula are shown in Table 3. It is worth noting that the search was conducted in July 2020.

**Table 3:** Results of the search in the considered databases.

Database	Search fields	Studies
Scopus	TITLE-ABS-KEY (Title, Abstract, and Keywords)	466
Web of Science	TS (Title, Abstract, and Keywords)	309
ERIC	Not specified. The query covered up the title, abstract and descriptors of the indexed documents.	239
Total		1014

### 4.3 Selection of the studies

**Fig 1:**
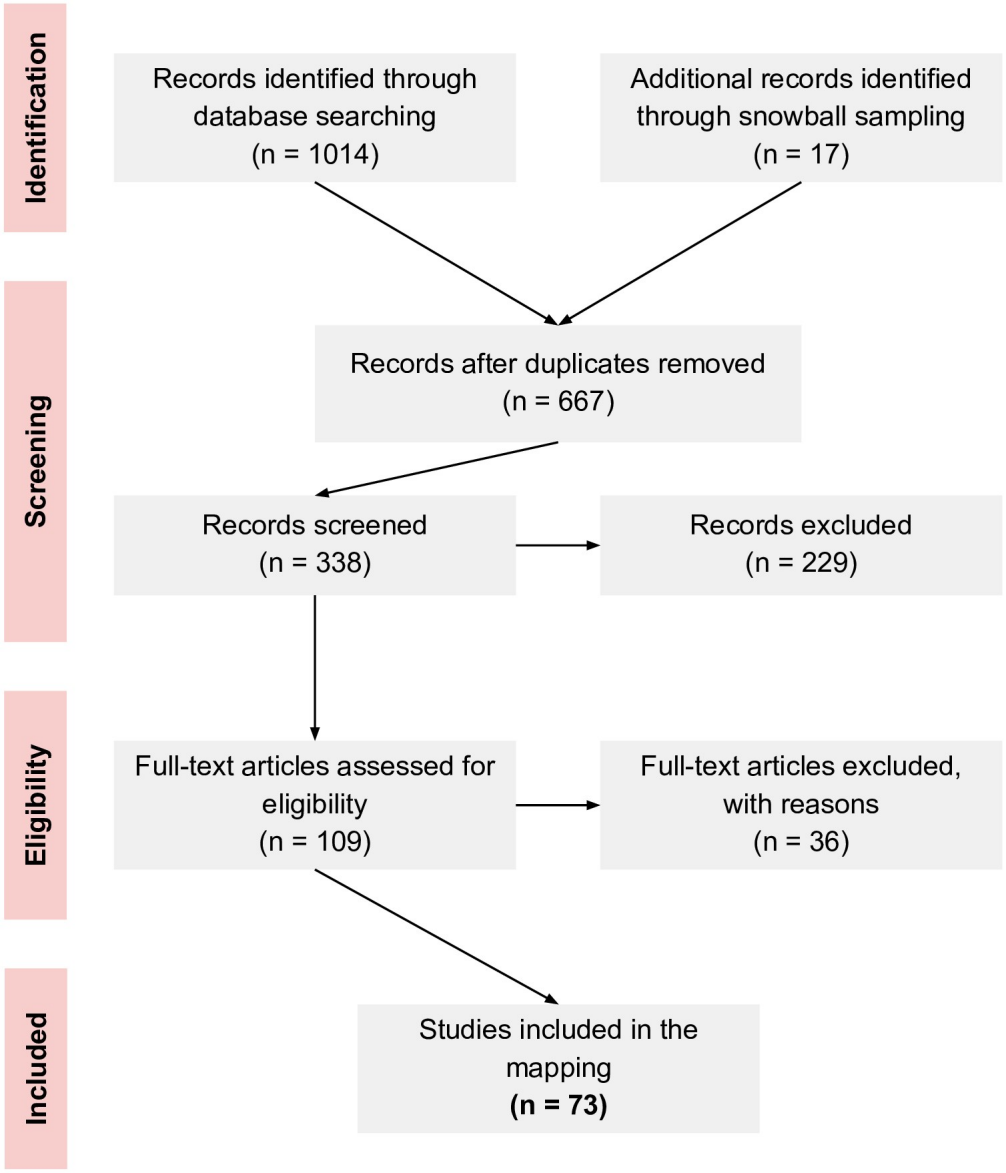
Study selection process.

During the selection, we followed several steps which are summarized in Fig 1. We considered the following as inclusion and exclusion criteria:

*Inclusion criteria*:

- Studies measuring digital competencies quantitatively in the context of higher education
- Studies published in the period of 2010 to July, 2020
- Studies published as journal articles

*Exclusion criteria*:

- Studies published in conference proceedings, book chapters
- Non-peer reviewed studies
- Studies published in books, technical reports, editorials
- Duplicates of other studies

The selection process was conducted by two authors independently, from the *Screening* step up to the *Eligibility* step (Fig 1). In the first case, the observed agreement and accounting for chance agreement were *p*_0_ = 0.928 and *κ* = 0.856 (p-value< 0.001), respectively. As for the *Eligibility* step, these values were *p*_0_ = 0.973 and *κ* = 0.938 (p-value< 0.001), respectively. In the case of the 36 studies rejected after reading their full texts, the particular reasons were as follows: 23 due to the exclusive use of qualitative data collection techniques, and 13 for not specifically assessing digital competencies.

### 4.4 Data extraction

Data extraction was conducted based on the template depicted in Table 4. As it can be observed, 10 variables related to both demographic and methodological features were considered. Regarding reliability and validity assessments, 4 different types were included for both groups (8 in total) [30]. For each type, we recorded the extent to which the study reported on the assessments, that is, *Not mentioned*, *Only mentioned*, *Referenced* (a previous study), and *Details are provided*. These dimensions were mapped into numerical values (0, 1, 2 and 3, respectively) in order to compute specific indicators for characterizing the studies’ reporting practices. Such indicators are described in the next section.

**Table 4:** Template used for data extraction.

Group	Variable	Dimension	Research question
Demographics	Year	2010,…,2020	RQ1,RQ3
	Continent	Africa, Asia, Europe, North America, Oceania, South America	
	Participant type	Undergraduate Students, Academic Staff, Post-graduate Students, Mixed	
	Discipline	Natural Sciences, Engineering, Health Sciences, Agricultural Sciences, Social Sciences, Humanities, Multidisciplinary	
	SJR quartile*	Q1,..,Q4, NQA (no quartile assigned)	
	JCR quartile**	Q1,..,Q4, NQA (no quartile assigned)	
Methodological features	Sample size	Small (less than 100), Medium (between 100 and 300), Large (over 300)	RQ1,RQ3
	Instrument source	Ad hoc, Proposed previously	
	Measured dimension	Knowledge, Skills, Attitudes, Several	
	Measurement form	Self-assessment, Objective, Both	
Reported reliability	Stability	Not mentioned (= 0), Only mentioned (= 1),	RQ2, RQ3
	Internal Consistency	Referenced (to a previous study) (= 2),	
	Equivalence	Details are provided (= 3)	
	Scalability		
Reported validity	Face validity	Not mentioned (= 0), Only mentioned (= 1),	RQ2, RQ3
	Content validity	Referenced (to a previous study) (= 2),	
	Criterion validity	Details are provided (= 3)	
	Construct validity		
Specific methods	Method	Specific methods used for reliability or validity assessments	RQ2
Dissemination	Citations	Number of citations received by the study from Google Scholar***	RQ2

*SCImago Journal Rank (https://www.scimagojr.com/),

**Journal Citation Reports (https://clarivate.com/),

***Google Scholar (https://scholar.google.com).

As in the selection process, data extraction was carried out by two authors in order to mitigate personal biases, especially when evaluating studies. As a result, the observed agreement and accounting for chance agreement were *p*_0_ = 0.965 and *κ* = 0.904 (p-value< 0.001), respectively.

### 4.5 Analysis and classification

Analysis and classification of the studies was carried out after data extraction. The obtained results were tabulated and visually summarized as shown in Sec. 5. A complete list of the reviewed studies and their corresponding classification according to the used data extraction template can be located at the following link https://osf.io/me36k.

In order to characterize the reporting practice of the studies, we defined three indicators, which are computed from the data extracted. They measure three different features of the studies. The first one, which we have called *External Coverage* represents how exhaustive the study is in conducting both reliability and validity assessments of any kind. Here, three cases are possible: 1) the study has not reported on any group of assessments, 2) the study reported on a single group (reliability or validity), and 3) the study reported on both groups. These three cases were labeled and numerically coded as *None* = 0.0, *Moderate* = 0.5 and *High* = 1.0, respectively.

Similarly, the second indicator, that we named *Internal Coverage*, is devoted to measure how comprehensive the study is inside the group of assessments it reported on. We have defined this indicator as the proportion between the number of assessment types conducted by the study and the number of possible assessments within the reported group or groups. Given that each group (reliability or validity) has 4 types of assessments, the total of possible assessments will be 4 if the study only reports on a single group, while it will be 8 if it reports on both groups at the same time. In turn, this indicator will range from 0 (conducting no assessment types at all) to 1 (conducting all assessment types withing the group or groups), with other values in between corresponding to several degrees of coverage.

The third indicator, *Reporting Depth*, measures how deep the study reports on the conducted assessments, that is, regardless of its external or internal coverage. It is computed as the normalized average from the study’s reporting levels achieved in the types of assessment that were conducted. These reporting levels are the ones defined in Table 4, which also have numerical codes. For example, a study conducting only one type of assessment with a reporting level of *Referenced* = 2 will have a *Reporting Depth* of 2/(1 ⋅ 3) = 0.667. Note that we divided by 1 ⋅ 3 because only one (1) type of assessment was conducted, while the maximum value a reporting level that may be achieved is 3 (corresponding to *Details are provided*). Therefore, this indicator ranges from 0 (no depth) to 1 (maximum depth). Of course, the latter case corresponds to the studies providing details in all of the conducted assessments.

From these indicators it is possible not only to rank the studies, but also to characterize their reporting practices. Note that this is necessary in order to answer research questions *RQ2.3*, *RQ3*, and *RQ3.1*. Here, three different approaches from multi-criteria decision analysis can be adopted [47]: 1) Full aggregation approach, 2) Outranking approach, or 3) Goal, Aspiration or Reference-level approach. Since, in our context, it is possible to certainly know both the *ideal* and the *anti-ideal* studies according to the three indicators we defined above, the third approach was adopted. Specifically, we applied the *Technique of Order Preference Similarity to the Ideal Solution* (TOPSIS) method [48], which assumes that the best alternative (study) is the one with the shortest distance to the ideal alternative and the furthest distance from the anti-ideal alternative [47]. Note that the ideal study is the one with *External Coverage* = *Internal Coverage* = *Reporting Depth* = 1. Contrarily, the anti-ideal study is the one with *External Coverage* = *Internal Coverage* = *Reporting Depth* = 0. Following the steps from the TOPSIS method [47], we computed the *Relative Closeness* coefficient for each study using euclidean distance and the same weights for the three indicators. This coefficient ranges from 0 to 1, where a value approaching 1 means that the study is close to the ideal study, while a value approaching 0 means the opposite. Conceptually, this ratio provides a good insight into how satisfactory the quality assessment reporting process was in the study.

**Fig 2:**
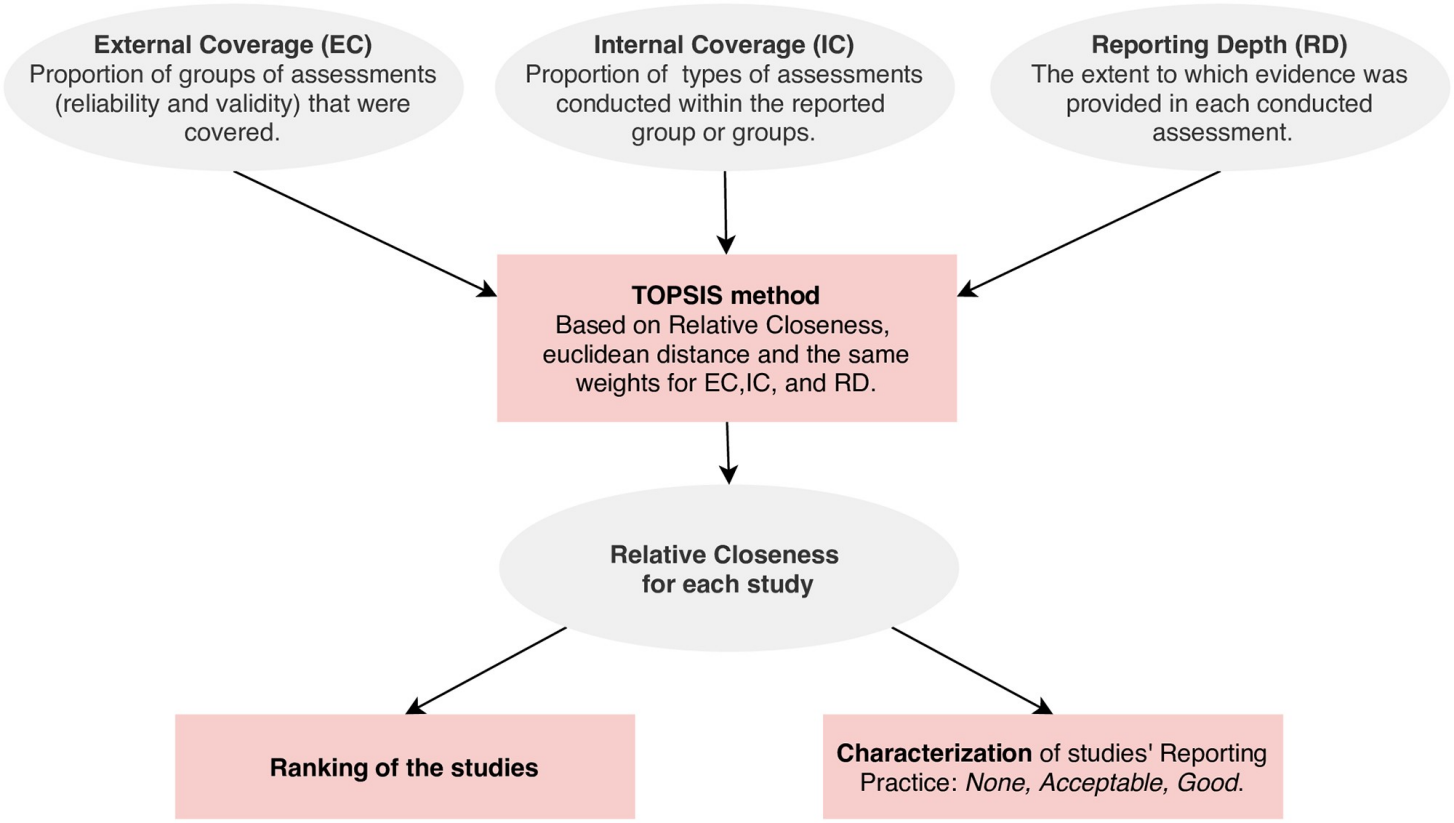
Process for ranking and characterizing studies’ reporting practices.

As noted above, *Relative Closeness* is, by definition, a continuous measure. Although this feature allows for convenient ordering of studies (to identify those with best practices), it is not entirely adequate to characterize studies. It would be more appropriate here to split the range of possible values of this measure into specific ranges associated to meaningful labels. Several alternatives can be adopted here (e.g., partitioning according to percentile ranges). For the sake of simplicity, we considered three classes (levels) for defining the variable *Reporting Practice* as follows:

$$\begin{array}{c} ReportingPractice\left(r_{i}\right)=\{\begin{array}{cc} None, & \text{if}\;r_{i}=0,\\ Acceptable, & \text{if}\;0<r_{i}\leq {\tilde{r}}_{>0},\\ Good & \text{if}\;r_{i}>{\tilde{r}}_{>0}. \end{array} \end{array}$$ (1)

where *r*_*i*_ is the relative closeness of study *i* and ${\tilde{r}}_{>0}=0.683$ is the midpoint of the interval [0.367, 1.000]. We have considered this particular interval because 0.367 and 1.000 are indeed the minimum and maximum values, respectively, that a study can achieve according to our definition of *Relative Closeness*. As a summary, Fig 2 illustrates how the proposed indicators contribute towards obtain both the ranking of the studies and the characterization of their reporting practice. Notice that this process is achieved through the TOPSIS method.

In addition to the above, we considered it fair to evaluate the studies from the point of view of their dissemination in the literature. In this sense, we have considered an indicator that we have called the *Dissemination Index*, which quantifies the degree to which the study has been cited by other works. Although this indicator does not accurately capture the real use of the instrument proposed by the study, it does give us an estimated idea of its impact on the scientific community. Formally, we have calculated this index as the rate of the number of citations of the study divided by the number of years it has been published. In this way, we seek to mitigate the possible advantage in the number of citations that studies published in earlier years may have over those published more recently. This index is a non-negative continuous magnitude with a minimum value of 0. A value close to 0 indicates that the study had low dissemination. The number of citations for each study was obtained from Google Scholar (scholar.google.com), while the calculation of the number of years was based on the year 2021. We selected Google Scholar for two reasons. The first is that this database has broad coverage (not only of the scientific literature, but also of the so-called gray literature) [49], and on the other hand, it serves as an independent reference to mitigate the bias of considering citations exclusively by the databases used in our systematic mapping study.

Finally, it is worth mentioning that, in order to find out what demographic and methodological features explain studies’ reporting practices (*RQ3.1*), we proceeded with an association analysis. Specifically, we conducted a Pearson’s Chi-squared test for testing whether each of the demographic and methodological variables are significantly associated with the *Reporting Practices*. We supplemented this analysis by calculating Cramér’s *V*, which is an effect size measurement for the Chi-square test [50]. Although other indicators could be equally effective in characterizing the strength of association (e.g., *V*^2^), we decided on this measure because it is intuitive and common in the context of association analysis of nominal categorical variables [50]. Further, for those significant associations (p-values below 0.05), we proceed with a post hoc analysis based on the standardized residuals of the Pearson’s Chi-squared test [51]. To address *RQ3.1*, we relied on an ordinal logistic regression model for describing *Reporting Practice* as a function of the variable *Year*. We have selected this regression model because of the nature of the variables involved.

### 4.6 Validity assessment

As suggested by [13], the validity of a systematic mapping study should be conducted by analyzing the main threats occurring in achieving the following validity types:

- *Descriptive validity*. The main thread here is that subjective studies have less descriptive validity than quantitative ones. However, our study is based in the count of data using a well-structured template (see Table 4). Consequently, we assumed that this threat is under control.
- *Theoretical validity*. Study selection and data extraction are important sources of threats affecting this validity type. However, we employed snowballing sampling (backward and forward) in order to mitigate the possibility of not including relevant studies. Additionally, authors reviewed each other’s steps in order to control the bias when selecting and extracting data. Regarding the quality of the sample of studies, it is clear that it is high since it comes from databases with great coverage of high quality venues.
- *Generalizability*. To assess this type of validity, we have to consider the internal and external generalizability of the results and methodology. In the first case, it is clear that results from this research can be generalized in the context of digital competence evaluations in higher education. So, internal generalizability is guaranteed. External generalizability is not guaranteed since digital competencies are not only measured in the context of higher education. Additionally, we have focused on quantitative instruments only, so our results describe an specific group of studies. With respect to the methodology, since systematic mapping studies and association analysis are general research methods, internal and external generalizability is guaranteed.
- *Interpretive validity*. In this case, threats may exist here because authors have worked together in previous research. So, it is possible that similar judgments when selecting and analyzing the primary studies arose.
- *Reproducibility*. We consider that reproducibility is guaranteed. This is because enough details are provided in this paper, that is, by following a systematic guide like the proposed by [13].

## 5 Results

In the following section, we present the results obtained from our analysis and classification of the studies. We organized them according to the research questions defined in Sec. 4.1.

### 5.1 Demographic and methodological features (RQ1)

**Fig 3:**
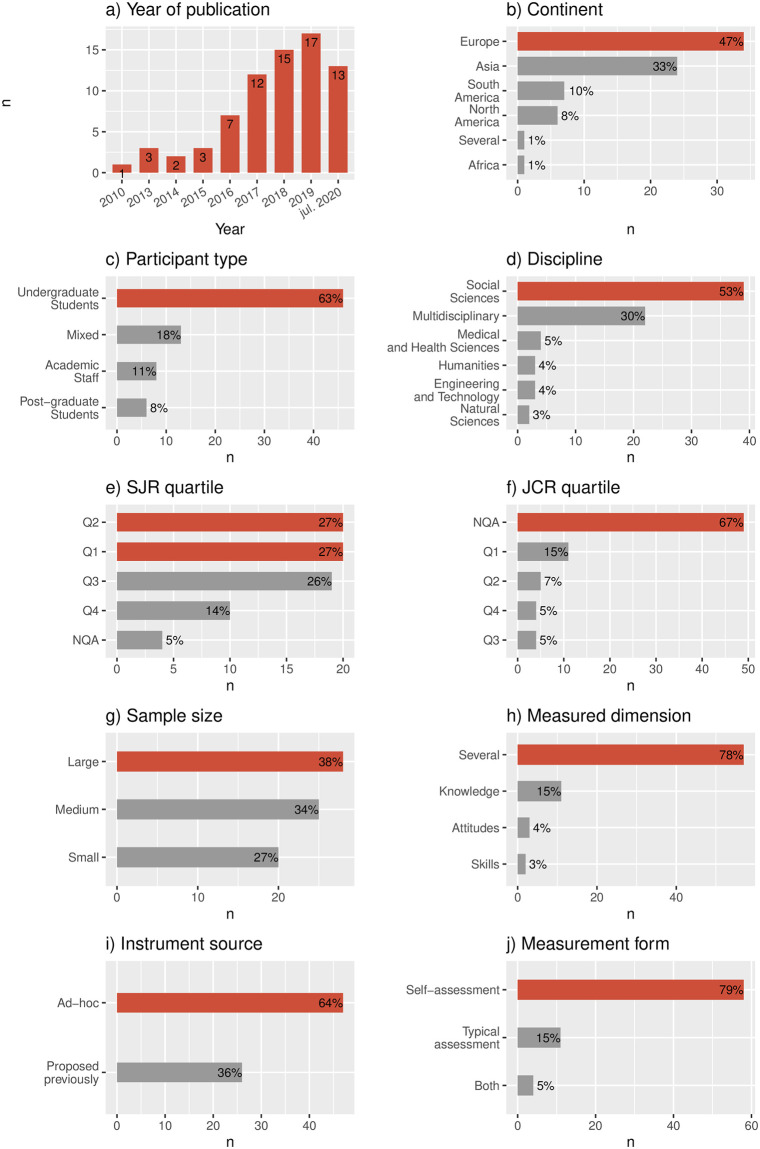
Demographic and methodological features of the studies.

Fig 3 summarizes the distribution of studies according to demographic features. More specifically, Fig 3a shows that evaluating digital competence in higher education is a research topic with an increasing number of studies over time. The special case of July 2020, which corresponds to the first half of 2020, contains 13 studies. A number that is clearly higher than half that of the previous year. Fig 3b and 3c indicate that most of the studies originate from Europe (47%), and are based on undergraduate students (63%) coming from Social Sciences programs (53%). Regarding the reputation of the journal, different results arise from the SJR and JCR indicators. While for the SJR, most of the studies have been published in venues with a quartile assigned (Fig 3e), the opposite occurs for the JCR indicator (Fig 3f). In the latter’s case, only 22% of the studies were published in top-tier journals (Q1 or Q2).

Taking into account the considered methodological features, Fig 3 reveals some interesting patterns. For instance, in regard to the sample size, Fig 3g shows that *Large* size samples are barely more frequent (38%) studied than the others. From Fig 3h it is clear that researchers have been focused in measuring several domains of digital competencies (78%) at the same time. More specifically, the employed instruments are mainly ad hoc (64%), that is, proposed by the authors of the study as shown in Fig 3i. According to Fig 3j, such measurements have been mostly self-assessments (79%), that is, based on participants perceptions about their own level of digital competence.

### 5.2 Reporting practices of quality assessments (RQ2)

**Fig 4:**
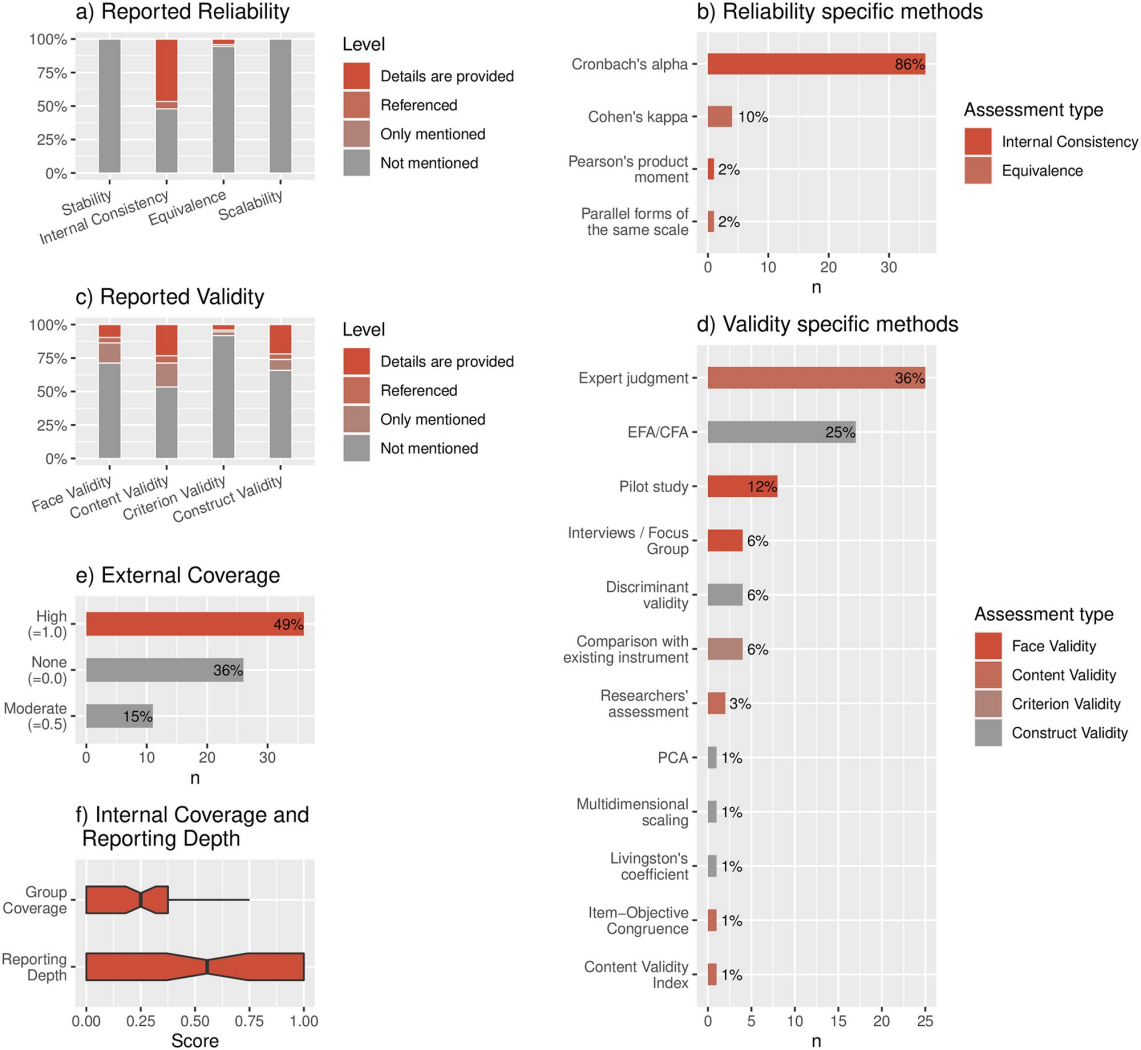
Reporting practices of quality assessments.

In this section, we answered several questions about how the studies reported on the quality of the employed instruments. To this end, Fig 4 summarizes the main results we obtained.

#### 5.2.1 What types of assessments and what specific methods are most often reported? (RQ2.1)

From Fig 4a, it is possible to observe that *Internal Consistency* is the most reported type of reliability assessment by far. About 50% of the studies provided enough details or referenced another study. Consistent with this result, Fig 4b shows that 86% of the studies conducting reliability assessments relied on Cronbach’s alpha, which is indeed a typical method for measuring internal consistency [30].

A different situation occurs with the validity assessments (Fig 4c). In this case, the studies are more evenly distributed. However, it is interesting to see that although *Content validity* is the most frequently reported assessment, about the half of the studies reporting on this provided no more detail than just mentioning that they did it. Fig 4d is more specific on how the assessment was conducted. *Expert judgment*, a typical method for content validity, is present in 36% of the studies conducting validity assessments. Interestingly, Exploratory Factor Analysis (EFA) and Confirmatory Factor Analysis (CFA) are present in 25% of the studies. They are used for conducting construct validity assessments.

#### 5.2.2 How comprehensive and how deep are these quality reports? (RQ2.2)

To characterize the reporting practices of the studies, we relied on the indicators described in Sec. 4.5. From Fig 4e, we can see that, in the case of *External Coverage*, 49% of the studies present a high coverage (reporting reliability and validity at the same time). However, 36% did not report on any group at all, while 15% did it moderately (that is, on a single group). Regarding the other two indicators, box plots from Fig 4f show that while more than 50% of studies achieve extreme values of *Reporting Depth* (0 or 1), in *Internal Coverage*, the maximum value is 0.75. In both cases, we see that at least the 25% of studies (1st Quartile) are equal to the minimum value of the 2nd Quartile, which is indeed 0. This is consistent with 36% of the studies with no *External Coverage*, as shown in Fig 4e. In fact, having no *External Coverage* at all implies no *Internal Coverage* and no *Reporting Depth*. So, the 36% of studies that are in this latter case correspond to those without any quality reporting practice at all.

#### 5.2.3 What studies achieve the best balance between coverage and depth in their reports? (RQ2.3)

Table 5 shows the studies arranged in descending order according to their *Relative Closeness* (the indicator we defined in Sec. 4.5). Also, note that in the first column, we have included the ordinal variable *Reporting Practice*, which allows for categorizing the studies through three groups: *None*, *Acceptable*, and *Good*. Studies belonging to the first group, which are also the ones with *Relative Closeness* = 0, were omitted from Table 5 due to space limitations. However, they can be accessed in the resource provided in Sec. 4.5.

**Table 5:** Studies ranked by their *Relative Closeness* to the ideal study in terms of reporting practices of quality assessments.

Reporting Practice	Study	Relative Closeness	External Coverage	Internal Coverage	Reporting Depth	Dissemination Index
Good	*[52]	0.805	1.000	0.625	1.000	32.500
	[53]	0.751	1.000	0.625	0.733	5.800
	[54]	0.750	1.000	0.500	1.000	3.000
	[55]	0.728	1.000	0.750	0.556	3.000
	*[56]	0.726	1.000	0.500	0.833	16.000
	[57]	0.726	1.000	0.500	0.833	2.500
	[58]	0.726	1.000	0.500	0.833	4.000
	[59]	0.707	1.000	0.500	0.750	0.000
	[60]	0.707	1.000	0.500	0.750	1.000
	[61]	0.707	1.000	0.500	0.750	2.000
	[62]	0.701	1.000	0.375	1.000	4.000
	*[63]	0.701	1.000	0.375	1.000	8.667
	[64]	0.701	1.000	0.375	1.000	6.000
	[65]	0.701	1.000	0.375	1.000	0.250
	*[66]	0.701	1.000	0.375	1.000	64.667
	[67]	0.701	1.000	0.375	1.000	7.200
	*[68]	0.701	1.000	0.375	1.000	8.000
	*[69]	0.684	1.000	0.500	0.667	14.667
	*[70]	0.684	1.000	0.500	0.667	11.600
	[71]	0.684	1.000	0.500	0.667	4.000
Acceptable	[72]	0.657	1.000	0.250	1.000	1.333
	*[73]	0.657	1.000	0.250	1.000	20.750
	*[74]	0.657	1.000	0.250	1.000	13.500
	[75]	0.657	1.000	0.250	1.000	7.333
	*[76]	0.657	1.000	0.250	1.000	16.500
	[77]	0.657	1.000	0.250	1.000	0.375
	[78]	0.657	1.000	0.250	1.000	7.000
	[79]	0.634	0.500	0.500	1.000	1.000
	[80]	0.634	0.500	0.500	1.000	7.333
	[81]	0.634	1.000	0.500	0.500	3.333
	*[82]	0.611	1.000	0.375	0.556	8.000
	*[83]	0.611	1.000	0.375	0.556	36.000
	[84]	0.611	1.000	0.375	0.556	3.000
	[85]	0.599	1.000	0.250	0.667	0.250
	[86]	0.599	1.000	0.250	0.667	0.750
	[87]	0.599	1.000	0.250	0.667	0.333
	*[88]	0.599	1.000	0.250	0.667	20.000
	[89]	0.599	1.000	0.250	0.667	3.286
	[90]	0.560	0.500	0.250	1.000	0.250
	[91]	0.560	0.500	0.250	1.000	7.000
	[92]	0.560	0.500	0.250	1.000	0.000
	[93]	0.446	0.500	0.500	0.333	7.333
	*[94]	0.367	0.500	0.250	0.333	12.250
	[95]	0.367	0.500	0.250	0.333	5.250
	[96]	0.367	0.500	0.250	0.333	0.000
	[97]	0.367	0.500	0.250	0.333	5.333
	[98]	0.367	0.500	0.250	0.333	0.000

*Studies with the highest dissemination level (dissemination index ≥8).

To complement this analysis, the rightmost column of Table 5 contains the dissemination index of the studies. It is easy to see that this characteristic allows us to classify the studies differently than Relative Closeness. In order to facilitate the identification of studies with high dissemination, we have divided the set of studies into tertiles. In the first one (T1) are the studies with dissemination index in the interval [0, 2.5], in the second (T2) those with values in the interval (2.5, 7.33], and in the third one (T3) those with the values in (7.33, 64.7]. Of course, those belonging to the T3 are the most widely disseminated studies of all. From these intervals, it is noteworthy that the distribution that the studies follow is not normal (e.g., it is highly skewed to the right), indicating that most of the studies have low dissemination rates compared to the highest value study (64.7). The 14 studies belonging to the latter category are marked with * in Table 5. Note that these studies have a dissemination index greater than or equal to 8.0 and would be, according to our analysis, those that achieve an adequate balance between quality and dissemination. To obtain an overview of how the studies are distributed according to these two characteristics, Table 6 summarizes the number for each variable level of *Reporting Practice* and *Level of Dissemination*. The results show a generally homogeneous distribution among the combinations of levels, although it is remarkable that the lowest number of studies (5) corresponds to those with *Good* reporting practices and *Low* levels of dissemination. A Chi-squared test confirms that there are no differences between these groups of studies (*χ*^2^ = 2.897, *df* = 4, p-value = 0.575).

**Table 6:** Distribution of studies according to their reporting practice and level of dissemination.

		Level of Dissemination	
Reporting Practice	*Low* (T1)	*Medium* (T2)	*High* (T3)
*None*	12	6	8
*Acceptable*	10	10	7
*Good*	5	8	7

### 5.3 What studies’ characteristics are more likely associated with certain reporting practices? (RQ3)

Table 7 shows the results obtained for an association analysis between *Reporting Practice* and studies’ demographic and methodological variables. Note that we also included the indicator of Cramér’s V for quantifying, in the range [0, 1], the strength of the association between variables. The larger this indicator, the stronger is the association between the variables. As Table 7 shows, only *Measured dimension* resulted in a significant association with *Reporting Practice* (p-value < 0.05). Cramér’s V indicates a low strength in this association (= 0.303).

**Table 7:** Pearson’s Chi-squared tests of independence between *Reporting Practice* and demographic and methodological variables.

Variable	*χ* ^2^	p-value	Cramér’s V
SJR quartile	8.086	0.439	0.235
JCR quartile	7.275	0.536	0.223
Continent	7.570	0.766	0.228
Discipline	12.017	0.290	0.287
Participant type	4.080	0.679	0.167
Sample size	1.913	0.772	0.115
Instrument source	0.844	0.663	0.107
Measured dimension	*13.432	0.021	0.303
Measurement form	2.722	0.649	0.137

*Value statistically significant (*α* = 0.05).

In order to find out which dimensions account for this significant association, we proceed with a post hoc analysis of the standardized residuals [51]. Table 8 summarized the results for this analysis. We observe that only one pair of dimensions was significantly associated (*Several* and *None*). This negative association means that studies focused on measuring several areas of digital competence are more likely to report on quality assessments.

**Table 8:** Standardized residuals from post hoc analyses on Pearson’s Chi-squared test.

		Reporting Practice		
Variable	Dimension	None	Acceptable	Good
Measured Dimension	Attitudes	2.378	-1.355	-1.087
	Knowledge	1.423	-1.402	-0.010
	Several	*-3.132	2.296	0.878
	Skills	1.928	-1.099	-0.881

*Value statistically significant (*α* = 0.05).

#### 5.3.1 How do quality reporting practices evolve over time? (RQ3.1)

Finally, we addressed the question of how studies’ reporting practices have evolved over the years. In this case, we estimated an ordered logistic regression model [99]. The corresponding model resulted in a significant coefficient for *Year* (= 0.279) and a standard error of 1.373*e* − 04. Brant’s test for checking the proportional odds assumption [100] gave a *χ*^2^ = 0.040 and a probability (p-value) equal to 0.850. Therefore, the relationship between each pair of outcome groups is the same under the coefficient estimate.

**Fig 5:**
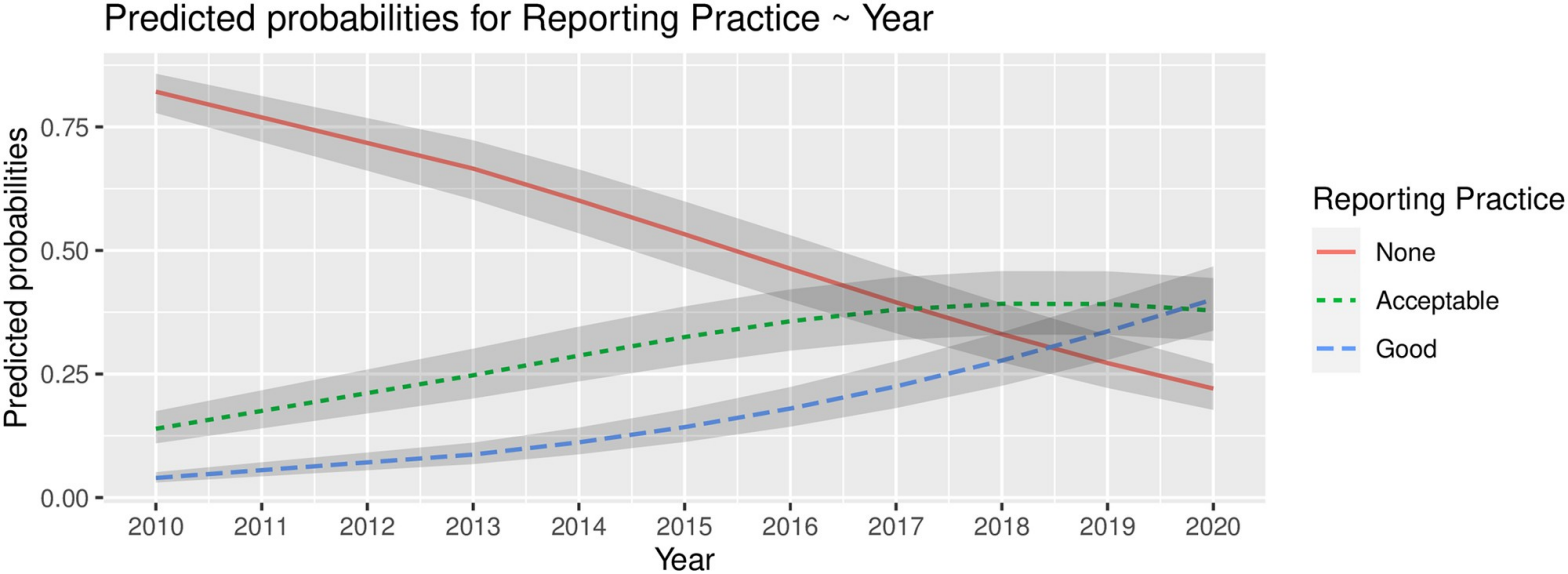
Predicted probabilities from an ordinal regression model with *Reporting Practice* as a function of *year*. The shaded ribbons correspond to the 95% confidence interval of the predicted values.

In order to better understand the effects of this coefficient, Fig 5 shows the predicted probabilities of each dimension of *Reporting Practice* as a function of the years. This plot shows a clear pattern in the evolution of the studies’ reporting practices. Note that the dominant dimension was *None* (no reporting at all) up to 2017. From this year up to the present (July 2020), *Acceptable* and *Good* reporting practices become more likely to appear in literature. We may also observe a decreasing trend over time in the predicted probabilities related to practice of no reporting quality assessments (*None*). On the contrary, predicted probabilities related to the practices of providing acceptable and good reports increase along the years. In the specific case of *Acceptable* reporting practices, the probabilities become steady from 2017 to the present.

## 6 Discussion and conclusion

From the results obtained, we observe that the number of studies evaluating digital competence in higher education has grown from 2010 to the present. This increasing behavior has been also reported by similar studies such as [11, 27]. Another important pattern arising from our study is the predominance of studies coming from Europe and which are based on undergraduate students from Social Sciences programs. This is somehow expected if we consider that evaluating digital competence in higher education is a research topic related to this discipline in this geographical area. So, these results suggest that researchers have been focused on studying what is perhaps the closest population to them: undergraduate students from Social Sciences. Other higher education actors from different disciplines, continents or with different roles (e.g., academic staff) have been less or not studied at all. This is consistent with the results reported in other similar studies [101], and this gap constitutes a clear opportunity for further research studying those underrepresented populations.

As a positive aspect, we observed most of the studies have been based on medium or large samples (involving more than 100 participants). Additionally, our results revealed that ad hoc questionnaires for measuring several dimensions of digital competencies were in the preference of the researchers. More specifically, the employed instruments mostly relied on the self-assessment of the participants. Although measuring several dimensions is a positive aspect of the studies, the high presence of self-assessment, ad hoc instruments is, from our viewpoint, a weakness in this field. In the case of ad hoc instruments, it would be a symptom of the lack of consensus on what digital competencies are in the context of higher education [11], or perhaps a consequence of divergent research purposes. In any case, this undermines the reuse of existing instruments, which indisputably affects the opportunity to improve and validate them on a large scale. As a related consequence, the reported results (even for populations with similar characteristics) are increasingly heterogeneous as time passes, making it difficult to draw general and precise conclusions from them.

With respect to the quality of the venue where the studies were published, different results from the SJR and JCR indicators were observed. While most studies appear in journals recognized by the SJR indicator, this is not the case for the JCR indicator. These differences are explained in part by the large coverage of SJR, as compared to that of JCR [102]. Since JCR is a more exclusive indicator, the results suggest that, under this indicator, most of the studies are published in low-tier journals.

In regard to the reported quality assessments, the obtained results are somewhat consistent with the use of questionnaires as data collection methods. It is for this reason that assessments of internal consistency (through Cronbach’s alpha), face validity (through pilot studies), content validity (through *Expert judgment*) and construct validity (through factor analysis) abound. Taking into account that, in this list, only internal consistency is a type of reliability assessment, it is clear that most studies report more types of validity than reliability assessments.

From the proposed indicators for appraising studies’ reporting practice, we observed that less than half of the studies conducted assessments from reliability and validity at the same time (*External Coverage*). In addition, the proportion of types of assessments conducted within each group (*Internal Coverage*), was lower than 50%, which indicates poor coverage. Regarding the depth in providing evidence supporting the quality assessments (*Reporting Depth*), the results showed that more than the half of the studies provided good levels. Overall, these results indicate that serious issues exist when conducting and reporting quality assessments of the employed instruments. It seems, however, that this is not a problem typical to digital competence evaluation in higher education, but a more general one in Educational Sciences. Studies such as that of [32] in the context of medical education and, more recently [41], in special education identified similar problems. Interestingly, we found no association between the degree of dissemination of the studies and their quality. This result indicates that studies with low quality in their reporting practices have statistically the same dissemination, in terms of citations, as those with acceptable or good quality.

The statistical analysis conducted, aimed at identifying what studies’ features could help to understand certain reporting practices, revealed just a few insights. We found only one significant, negative association between measuring several digital competence areas at the same time, and no reporting of any quality assessment at all. This suggests that those studies devoted to measuring several areas of digital competence are more likely to report on quality assessments of the employed instruments. Although in part explained by the lack of enough data, the absence of significant associations in the case of the other variables is a warning sign. For instance, we might expect that those studies published in top-tier venues are more likely to exhibit good reporting practices. In the same line, we would like to see more concern in conducting, and hence, reporting quality assessments by those studies using ad hoc, self-assessment instruments. However, no evidence was found supporting these beliefs. The good news is that, according to our estimates, the tendency to report more and with better quality evaluations of the instruments used to measure digital competencies in higher education is growing over time. Although slight, this growth is significant.

In summary, it is clear that, based on the collected evidence, we can not trust in all of the available instruments published till date. The list of studies we provided, which rank them according to their degree of coverage and depth in reporting quality assessments, is expected to help researchers and practitioners in identifying relevant instruments to advance in the field.

## 7 Implications

The results obtained by our study have important implications from both practical and research perspectives. First, our demographic and methodological characterization of the studies implies that practitioners and policymakers have a rich body of prior experience that can be taken into account when measuring digital competencies in higher education settings. Similarly, researchers have clear opportunities for future research. The evaluation of less studied populations (e.g., university professors from regions such as South America, North America or Africa) or validating existing instruments through comparative studies, are two examples of these opportunities.

Second, the fact that more than half of the studies do not conduct reliability and validity assessments at the same time, and those that do, cover less than half of the criteria within each category, implies that the selection of an existing instrument to measure digital competencies in higher education should be done with care. From a research perspective, this result implies that more emphasis should be placed on ensuring that the instruments used meet adequate levels of reliability and validity. Research opportunities exist in this context, especially related to the development of validation studies that are based on proposed instruments with little evidence of quality assessments.

Third, the fact that the quality and dissemination of the studies do not correlate is a warning sign regarding the selection of the most appropriate instruments in higher education. This implies that the practitioner, as well as the researcher, should not be guided purely by the quantity of citations of the study, but also the quality of the instrument in terms of the psychometric properties that we have considered in this paper. In the same line, our results imply that the selection of instruments based on demographic or methodological similarities is not reliable. Therefore, emphasis should be placed on the specific evidences reported by the studies.

Fourth, the evolution of studies towards the inclusion of a greater number of quality assessments has as its main implication that, in the near future, it should be easier to find better validated instruments to measure digital competencies in higher education. This augurs a favorable scenario for practitioners and researchers. However, much remains to be done. The great heterogeneity of approaches to evidence the quality of a quantitative instrument is a clear indication of the absence of a “standard” in this field of research. Future research could focus on proposing, for each measurement scenario, which methods should be applied to ensure the reliability and validity of the instrument under consideration.

In a more general context, the fact that academia is placing increasing interest in measuring digital competencies is a good sign that awareness is growing of the importance of understanding and monitoring the achievement of the UN Sustainable Development Goals (SDGs) [103]. As precisely mentioned in [104], ICTs are considered key catalysts for the achievement of the 17 SGDs. In this context, our results contribute to raising awareness of the importance of correctly measuring digital competencies in higher education, a key step to know how far we have progressed and what still needs to be done.

## 8 Limitations

Regardless of the relevance of the results obtained, this research has important limitations. First, we based our results on a limited sample extracted only from journal articles that were published in the last 10 years. Therefore, we do not know the extent to which these results also apply to studies published in different venues and on different dates. Similarly, our study is limited by the variables used for the characterization of the studies. Therefore, there is a possibility that other variables (not included here) not only better describe the studies, but also more appropriately explain the presence of certain reporting practices.

Another important limitation is that we have evaluated the studies according to the practices and levels (depth) with which they report quality assessments. Therefore, in no way do our results indicate which instruments are more appropriate for measuring digital competence in higher education. We are aware that this is a much more complex task which depends on several factors including context. Therefore, in the future, it will be necessary to develop more research to answer these and other related scientific questions. In this sense, an important question is, “How do qualitative data collection techniques guarantee the quality of measurement?” Our future research will focus on providing answers to these issues.

## References

[1] Konsbruck Robert L. Impacts of information technology on society in the new century; 2002. [Cited 12 December 2020]. Available from: https://www.zurich.ibm.com/pdf/news/Konsbruck.pdf.

[2] Makridakis S. The forthcoming Artificial Intelligence (AI) revolution: Its impact on society and firms. Futures. 2017;90:46–60. doi: 10.1016/j.futures.2017.03.006

[3] Ferrari A. Digital Competence in Practice: An Analysis of Frameworks. Institute for Prospective Technological Studies (Joint Research Centre); 2012. Available from: https://op.europa.eu/s/pcz6.

[4] Gallardo-Echenique EE, de Oliveira JM, Marqués L, Esteve-Mon F. Digital competence in the knowledge society. Journal of Online Learning and Teaching. 2015;11(1):1.

[5] Krumsvik RJ. Teacher educators’ digital competence. Scandinavian Journal of Educational Research. 2014;58(3):269–280. doi: 10.1080/00313831.2012.726273

[6] Ala-Mutka K, Punie Y, Redecker C. Digital competence for lifelong learning. European Commission, Joint Research Centre, Institute for Prospective Technological Studies; 2008.

[7] Mueller RO, Knapp TR. Reliability and validity. In: Hancock GR, Stapleton LM, Mueller RO, editors. The Reviewer’s Guide to Quantitative Methods in the Social Sciences. 2nd ed. Routledge; 2019. p. 397–401.

[8] Prendes MP, Gutiérrez I, Martínez F. Competencia digital: una necesidad del profesorado universitario en el siglo XXI. Revista de Educación a Distancia (RED). 2018;(56).

[9] Palacios Hidalgo FJ, Gómez Parra ME, Huertas Abril CA. Digital and Media Competences: Key Competences for EFL Teachers. Teaching English with Technology. 2020;20(1):43–59.

[10] Ilomäki L, Paavola S, Lakkala M, Kantosalo A. Digital competence—An emergent boundary concept for policy and educational research. Education and Information Technologies. 2014;21(3):655–679.

[11] Spante M, Hashemi SS, Lundin M, Algers A. Digital competence and digital literacy in higher education research: Systematic review of concept use. Cogent Education. 2018;5(1):1–21. doi: 10.1080/2331186X.2018.1519143

[12] Pettersson F. On the issues of digital competence in educational contexts—a review of literature. Education and Information Technologies. 2018;23(3):1005–1021. doi: 10.1007/s10639-017-9649-3

[13] Petersen K, Vakkalanka S, Kuzniarz L. Guidelines for conducting systematic mapping studies in software engineering: An update. Information and Software Technology. 2015;64:1–18. doi: 10.1016/j.infsof.2015.03.007

[14] Tsankov N, Damyanov I. Education Majors’ Preferences on the Functionalities of E-Learning Platforms in the Context of Blended Learning. International Journal of Emerging Technologies in Learning (iJET). 2017;12(05):202. doi: 10.3991/ijet.v12i05.6971

[15] Johnson L, Levine A, Smith R, Stone S. The 2010 Horizon Report. Austin, Texas: The New Media Consortium; 2010. Available from: https://files.eric.ed.gov/fulltext/ED510220.pdf.

[16] Ferrari A. DIGCOMP: A Framework for Developing and Understanding Digital Competence in Europe. Eur 26035 ed. Luxembourg: Publications Office of the European Union; 2013.

[17] Vuorikari R, Punie Y, Carretero S, Van Den Brande L. DigComp 2.0: The Digital Competence Framework for Citizens. Update Phase 1: The Conceptual Reference Model. Eur 27948 en ed. June. Luxembourg: Publication Office of the European Union; 2016.

[18] Carretero S, Vuorikari R, Punie Y. DigComp 2.1: The Digital Competence Framework for Citizens with eight proficiency levels and examples of use. Eur 28558 en ed. Luxembourg: Publications Office of the European Union; 2017.

[19] Henríquez Coronel PM, Gisbert Cervera M, Fernández Fernández I. La evaluación de la competencia digital de los estudiantes una revisión al caso latinoamericano. Chasqui: Revista Latinoamericana de Comunicación. 2018;(137):93–112.

[20] Lopes Pereira N, Aisenberg Ferenhof H, Spanhol FJ. Strategies for the management of digital competences in higher education: A review in the literature. RELATEC: Revista Latinoamericana de Tecnología Educativa. 2019;18(1):71–90.

[21] Rivas RS, Novoa-Hernández P, Rodríguez RS. Evaluation of the presence of digital competences in higher education institutions. RISTI—Revista Iberica de Sistemas e Tecnologias de Informacao. 2019;2019(E21):23–36.

[22] Gibson PF, Smith S. Digital literacies: Preparing pupils and students for their information journey in the twenty-first century. Information and Learning Science,. 2018.

[23] Reis C, Pessoa T, Gallego-Arrufat MJ. Literacy and digital competence in Higher Education: A systematic review. REDU Revista de Docencia Universitaria. 2019;17(1):45–58.

[24] de Paulo Moura KM. Systematic review on digital literacy in teacher training. Texto Livre: Linguagem e Tecnologia. 2019;12(3):128–143.

[25] Lopez Nuñez JA, Campos Soto MN, Aznar Diaz I, Rodriguez Jimenez C. Digital competence of the teaching staff to attend to students with learning difficulties. A theoretical review. Revista Electrónica Interuniversitaria de Formación del Profesorado. 2020;23(2):143–154.

[26] Ocana-Fernandez Y, Valenzuela-Fernandez L, Morillo-Flores J. The Digital Competence in the University Teacher. Propósitos y representaciones. 2020;8(1):1–13.

[27] Sanchez-Caballe A, Gisbert-Cervera M, Esteve-Mon F. The digital competence of university students: A systematic literature review. Aloma. 2020;38(1):63–74. doi: 10.51698/aloma.2020.38.1.63-74

[28] Bandalos DL. Measurement Theory and Applications for the Social Sciences. Methodology in the Social Sciences. Guilford Publications; 2018.

[29] Scholtes VA, Terwee CB, Poolman RW. What makes a measurement instrument valid and reliable? Injury. 2011;42(3):236–240. 21145544 10.1016/j.injury.2010.11.042

[30] Bannigan K, Watson R. Reliability and validity in a nutshell. Journal of Clinical Nursing. 2009;18(23):3237–3243. doi: 10.1111/j.1365-2702.2009.02939.x 19930083

[31] Ratanawongsa N, Thomas PA, Marinopoulos SS, Dorman T, Wilson LM, Ashar BH, et al. The Reported Validity and Reliability of Methods for Evaluating Continuing Medical Education: A Systematic Review. Academic Medicine. 2008;83(3):274–283. doi: 10.1097/ACM.0b013e3181637925 18316877

[32] Speyer R, Pilz W, Kruis JVD, Brunings JW. Reliability and validity of student peer assessment in medical education: A systematic review. Medical Teacher. 2011;33(11):e572–e585. doi: 10.3109/0142159X.2011.610835 22022910

[33] Mushtaq N, Beebe LA. A review of the validity and reliability of smokeless tobacco dependence measures. Addictive Behaviors. 2012;37(4):361–366. doi: 10.1016/j.addbeh.2011.12.003 22244704

[34] Golijani-Moghaddam N, Hart A, Dawson DL. The Implicit Relational Assessment Procedure: Emerging reliability and validity data. Journal of Contextual Behavioral Science. 2013;2(3-4):105–119. doi: 10.1016/j.jcbs.2013.05.002

[35] Olivari MG, Tagliabue S, Confalonieri E. Parenting Style and Dimensions Questionnaire: A Review of Reliability and Validity. Marriage & Family Review. 2013;49(6):465–490. doi: 10.1080/01494929.2013.770812

[36] Radakovic R, Harley C, Abrahams S, Starr JM. A systematic review of the validity and reliability of apathy scales in neurodegenerative conditions. International Psychogeriatrics. 2014;27(6):903–923. doi: 10.1017/S1041610214002221 25355282

[37] Navas-Ferrer C, Urcola-Pardo F, Subirón-Valera AB, Germán-Bes C. Validity and Reliability of Objective Structured Clinical Evaluation in Nursing. Clinical Simulation in Nursing. 2017;13(11):531–543. doi: 10.1016/j.ecns.2017.07.003

[38] Knobel P, Dadvand P, Maneja-Zaragoza R. A systematic review of multi-dimensional quality assessment tools for urban green spaces. Health & Place. 2019;59:102198. doi: 10.1016/j.healthplace.2019.102198 31525616

[39] Delatorre MZ, Wagner A. Marital Quality Assessment: Reviewing the Concept, Instruments, and Methods. Marriage & Family Review. 2020;56(3):193–216. doi: 10.1080/01494929.2020.1712300

[40] Mirza N, Cinel J, Noyes H, McKenzie W, Burgess K, Blackstock S, et al. Simulated patient scenario development: A methodological review of validity and reliability reporting. Nurse Education Today. 2020;85:104222. doi: 10.1016/j.nedt.2019.104222 31783266

[41] Lee SH, Kim YR, Park J. A review of instruments measuring special educators’ competencies. British Journal of Special Education,. 2020. doi: 10.1111/1467-8578.12315

[42] Grant MJ, Booth A. A typology of reviews: An analysis of 14 review types and associated methodologies. Health Information & Libraries Journal. 2009;26(2):91–108. doi: 10.1111/j.1471-1842.2009.00848.x 19490148

[43] Banerjee M, Capozzoli M, McSweeney L, Sinha D. Beyond kappa: A review of interrater agreement measures. Canadian Journal of Statistics. 1999;27(1):3–23. doi: 10.2307/3315487

[44] Kates AW, Wu H, Coryn CLS. The effects of mobile phone use on academic performance: A meta-analysis. Computers & Education. 2018;127:107–112. doi: 10.1016/j.compedu.2018.08.012

[45] Murillo GG, Novoa-Hernández P, Rodríguez RS. Technology Acceptance Model and Moodle: A systematic mapping study. Information Development. 2020; p. 1–16.

[46] Higgins JPT, Thomas J, Chandler J, Cumpston M, Li T, Page MJ, et al. Cochrane Handbook for Systematic Reviews of Interventions. Wiley Cochrane Series. Wiley; 2019. 10.1002/14651858.ED000142 PMC10284251 31643080

[47] Ishizaka A, Nemery P. Multi-Criteria Decision Analysis. John Wiley & Sons Ltd; 2013.

[48] Behzadian M, Khanmohammadi Otaghsara S, Yazdani M, Ignatius J. A state-of the-art survey of TOPSIS applications. Expert Systems with Applications. 2012;39(17):13051–13069. doi: 10.1016/j.eswa.2012.05.056

[49] Martín-Martín A, Orduna-Malea E, Thelwall M, López-Cózar ED. Google Scholar, Web of Science, and Scopus: A systematic comparison of citations in 252 subject categories. Journal of Informetrics. 2018;12(4):1160–1177. doi: 10.1016/j.joi.2018.09.002

[50] Kvålseth TO. Association Measures for Nominal Categorical Variables. In: Lovric M, editor. International Encyclopedia of Statistical Science. Berlin, Heidelberg: Springer Berlin Heidelberg; 2011. p. 61–64. Available from: 10.1007/978-3-642-04898-2_122.

[51] Sharpe D. Chi-Square Test is Statistically Significant: Now What? Practical Assessment, Research, and Evaluation. 2015;20(8):1–10.

[52] Lázaro-Cantabrana JL, Usart-Rodríguez M, Gisbert-Cervera M. Assessing Teacher Digital Competence: the Construction of an Instrument for Measuring the Knowledge of Pre-Service Teachers. Journal of New Approaches in Educational Research. 2019;8(1):73–78. doi: 10.7821/naer.2019.1.370

[53] Hallaq T. Evaluating online media literacy in higher education: Validity and reliability of the Digital Online Media Literacy Assessment (DOMLA). Journal of Media Literacy Education. 2016;8(1):62–84.

[54] Romero Martínez SJ, Ordóñez Camacho XG, Guillén-Gamez FD, Agapito JB. Attitudes toward technology among distance education students: Validation of an explanatory model. Online Learning Journal. 2020;24(2):59–75.

[55] Gallego-Arrufat MJ, Torres-Hernández N, Pessoa T. Competence of future teachers in the digital security area. Comunicar. 2019;27(61):53–62. doi: 10.3916/C61-2019-05

[56] Maderick JA, Zhang S, Hartley K, Marchand G. Preservice teachers and self-assessing digital competence. Journal of Educational Computing Research. 2015;54(3):326–351. doi: 10.1177/0735633115620432

[57] Suwanroj T, Leekitchwatana P, Pimdee P. Confirmatory factor analysis of the essential digital competencies for undergraduate students in Thai higher education institutions. Journal of Technology and Science Education. 2019;9(3):340–356. doi: 10.3926/jotse.645

[58] Tourón J, Martín D, Asencio EN, Pradas S, íñigo V. Validación de constructo de un instrumento para medir la competencia digital docente de los profesores (CDD). Revista Española de Pedagogía. 2018;76(269).

[59] Arango DAG, Fernández JEV, Rojas ÓAC, Gutiérrez CAE, Villa CFH, Grisales MAB. Digital competence in university teachers: Evaluation of relation between attitude, training and digital literacy in the use of ict in educational environments. RISTI—Revista Iberica de Sistemas e Tecnologias de Informacao. 2020;2020(E29):538–552.

[60] Barišić KD, Divjak B, Kirinić V. Education systems as contextual factors in the technological pedagogical content knowledge framework. Journal of Information and Organizational Sciences. 2019;43(2):163–183. doi: 10.31341/jios.43.2.3

[61] Moreno D, Palacios A, Barreras Á, Pascual V. An assessment of the impact of teachers’ digital competence on the quality of videos developed for the flipped math classroom. Mathematics. 2020;8(2):148. doi: 10.3390/math8020148

[62] Alt D, Raichel N. Enhancing perceived digital literacy skills and creative self-concept through gamified learning environments: Insights from a longitudinal study. International Journal of Educational Research. 2020;101. doi: 10.1016/j.ijer.2020.101561

[63] Eger L, Klement M, Tomczyk Ł, Pisoňová M, Petrová G. Different user groups of university students and their ICT competence: Evidence from three countries in Central Europe. Journal of Baltic Science Education. 2018;17(5):851–866. doi: 10.33225/jbse/18.17.851

[64] González-Martínez J, Esteve-Mon FM, Rada VL, Vidal CE, Cervera MG. Incotic 2.0. A new self-assessment tool for digital competences at the university studies. Profesorado. 2018;22(4):133–152.

[65] Gutiérrez-Castillo JJ, Cabero-Almenara J, Estrada-Vidal LI. Design and validation of an instrument for evaluation of digital competence of University student [Diseño y validación de un instrumento de evaluación de la competencia digital del estudiante universitario]. Espacios. 2017;38(10):16.

[66] McGrew S, Breakstone J, Ortega T, Smith M, Wineburg S. Can students evaluate online sources? Learning from assessments of civic online reasoning. Theory and Research in Social Education. 2018;46(2):165–193. doi: 10.1080/00933104.2017.1416320

[67] Phuapan P, Viriyavejakul C, Pimdee P. An analysis of digital literacy skills among Thai university seniors. International Journal of Emerging Technologies in Learning. 2016;11(3):24–31. doi: 10.3991/ijet.v11i03.5301

[68] Silva J, Usart M, Lázaro-Cantabrana JL. Teacher’s digital competence among final year Pedagogy students in Chile and Uruguay. Comunicar. 2019;27(61):31–40. doi: 10.3916/C61-2019-03

[69] Blayone TJB, Mykhailenko O, VanOostveen R, Barber W. Ready for digital learning? A mixed-methods exploration of surveyed technology competencies and authentic performance activity. Education and Information Technologies. 2018;23(3):1377–1402. doi: 10.1007/s10639-017-9662-6

[70] Cózar-Gutiérrez R, de Moya-Martínez MV, Hernández-Bravo JA, Hernández-Bravo JR. Knowledge and use of information and communications technology (ICT) by prospective teachers according to their learning styles. Formacion Universitaria. 2016;9(6):105–118.

[71] Phan TC, Ngo TT, Phan TM. Assessment of information technology use competence for teachers: Identifying and applying the information technology competence framework in online teaching. Journal of Technical Education and Training. 2020;12(1 Special Issue):149–162.

[72] Gómez Trigueros IM. New learning of Geography with technology: The TPACK model. European Journal of Geography. 2018;9(1):38–48.

[73] Guzmán-Simón F, García-Jiménez E, López-Cobo I. Undergraduate students’ perspectives on digital competence and academic literacy in a Spanish university. Computers in Human Behavior. 2017;74:196–204. doi: 10.1016/j.chb.2017.04.040

[74] He T, Li S. A comparative study of digital informal learning: The effects of digital competence and technology expectancy. British Journal of Educational Technology,. 2019. doi: 10.1111/bjet.12778

[75] Kim HJ, Hong AJ, Song HD. The relationships of family, perceived digital competence and attitude, and learning agility in sustainable student engagement in higher education. Sustainability (Switzerland). 2018;10(12):4635. doi: 10.3390/su10124635

[76] McGrew S, Smith M, Breakstone J, Ortega T, Wineburg S. Improving university students’ web savvy: An intervention study. British Journal of Educational Psychology. 2019;89(3):485–500. doi: 10.1111/bjep.12279 30993684

[77] Moreno GC, Delgado SC. Evaluación de la competencia digital y las actitudes hacia las tic del alumnado universitario. Revista de Investigación Educativa. 2013;31(2).

[78] Rubilar PS, Alveal FR, Fuentes ACM. Evaluation of digital and pedagogical literacy in ICT based on the opinions of initial teacher education students [Evaluación de la alfabetización digital y pedagógica en TIC, a partir de las opiniones de estudiantes en formación inicial docente]. Educacao e Pesquisa. 2017;43(1):127–143.

[79] Flores AZ, Gurieva N, Arredondo VHJ. The holistic practice of educator digital competencies: Diagnostics and prospective. Pensamiento Educativo. 2020;57(1).

[80] Hong AJ, Kim HJ. College students” Digital Readiness for Academic Engagement (DRAE) Scale: Scale development and validation. Asia-Pacific Education Researcher. 2018;27(4):303–312. doi: 10.1007/s40299-018-0387-0

[81] Pieterse E, Greenberg R, Santo Z. A multicultural approach to digital information literacy skills evaluation in an Israeli college. Communications in Information Literacy. 2018;12(2):107–127. doi: 10.15760/comminfolit.2018.12.2.4

[82] Basantes-Andrade A, Cabezas-González M, Casillas-Martín S. Digital competences relationship between gender and generation of university professors. International Journal on Advanced Science, Engineering and Information Technology. 2020;10(1):205–211. doi: 10.18517/ijaseit.10.1.10806

[83] Casillas Martín S, Cabezas González M, García Peñalvo FJ. Digital competence of early childhood education teachers: attitude, knowledge and use of ICT. European Journal of Teacher Education. 2020;43(2):210–223. doi: 10.1080/02619768.2019.1681393

[84] González MC, Martín SC. Social educators: A study of digital competence from a gender differences perspective. Croatian Journal of Education. 2018;20(1):11–42.

[85] Cabezas González M, Casillas Martín S. Are future social educators digital residents? [¿Son los futuros educadores sociales residentes digitales?]. Revista Electrónica de Investigación Educativa. 2017;19(4):61–72.

[86] Cabezas González M, Casillas Martín S, Sanches-Ferreira M, Teixeira Diogo FL. Do gender and age affect the level of digital competence? A study with university students. FONSECA-Journal of Communication. 2017;(15):115–132.

[87] Harati A, Rahmatizadeh S, Valizadeh-Haghi S. Allied medical sciences students’ experiences with technology: Are they digitally literate? Library Philosophy and Practice. 2018;2018.

[88] Miguel-Revilla D, Martínez-Ferreira JM, Sánchez-Agustí M. Assessing the digital competence of educators in social studies: An analysis in initial teacher training using the TPACK-21 model. Australasian Journal of Educational Technology,. 2020. doi: 10.14742/ajet.5281

[89] Pérez-Mateo-Subirà M, Romero-Carbonell M, Romeu-Fontanillas T. Collaborative construction of a project as a methodology for acquiring digital competences. Comunicar. 2014;21(42):15–24. doi: 10.3916/C42-2014-01

[90] Agustin LD, Carlos JPJ, Arturo TGC, Alicia FGM. Study about the perception of basic digital competences of students of a Chilean university. Turkish Online Journal of Educational Technology. 2017;2017(November Special Issue INTE):1023–1029.

[91] Guillén-Gámez FD, Peña MP. Univariate analysis of digital competence in physical education: An empirical study. Retos. 2020;37:326–332.

[92] Jarad GA, Shaalan MA. Assessment of digital competence of employees and teaching staff at the Technical College of Management—Kufa. International Journal of Innovation, Creativity and Change. 2020;12(12):1027–1043.

[93] Thorell M, Fridorff-Jens PK, Lassen P, Lange T, Kayser L. Transforming students into digital academics: A challenge at both the individual and the institutional level approaches to teaching and learning. BMC Medical Education. 2015;15(1). doi: 10.1186/s12909-015-0330-5 25890174 PMC4377857

[94] García-Martín J, García-Sánchez JN. Pre-service teachers’ perceptions of the competence dimensions of digital literacy and of psychological and educational measures. Computers and Education. 2017;107:54–67. doi: 10.1016/j.compedu.2016.12.010

[95] Mehran P, Alizadeh M, Koguchi I, Takemura H. Are Japanese digital natives ready for learning english online? A preliminary case study at Osaka University. International Journal of Educational Technology in Higher Education. 2017;14(1). doi: 10.1186/s41239-017-0047-0

[96] Naim S, Razak NA. Effect of personal and professional characteristics towards ESL lecturers’ digital competence. International Journal of Advanced Science and Technology. 2020;29(4):1029–1049.

[97] Saxena P, Gupta SK, Mehrotra D, Kamthan S, Sabir H, Katiyar P, et al. Assessment of digital literacy and use of smart phones among Central Indian dental students. Journal of Oral Biology and Craniofacial Research. 2018;8(1):40–43. doi: 10.1016/j.jobcr.2017.10.001 29556462 PMC5854558

[98] Sundararasan T, Kalaiyarasan G, Udhaya Mohan Babu R, Arockia Anita X, Sarkar SR. Digital literacy program to undergraduate students through priceless laptop scheme: An illuminative evaluation. International Journal of Advanced Science and Technology. 2019;28(18):592–599.

[99] Harrell FE. Ordinal Logistic Regression. In: Regression Modeling Strategies: With Applications to Linear Models, Logistic and Ordinal Regression, and Survival Analysis. Cham: Springer International Publishing; 2015. p. 311–325.

[100] Brant R. Assessing Proportionality in the Proportional Odds Model for Ordinal Logistic Regression. Biometrics. 1990;46(4):1171–1178. doi: 10.2307/2532457 2085632

[101] Granić A, Marangunić N. Technology acceptance model in educational context: A systematic literature review. British Journal of Educational Technology. 2019;50(5):2572–2593. doi: 10.1111/bjet.12864

[102] Delgado-López-Cózar E, Cabezas-Clavijo Á. Ranking journals: Could Google Scholar Metrics be an alternative to Journal Citation Reports and Scimago Journal Rank? Learned Publishing. 2013;26(2):101–113. doi: 10.1087/20130206

[103] SDGs. The sustainable development goals report 2019. United Nations publication issued by the Department of Economic and Social Affairs; 2019. Available from: https://undocs.org/E/2019/68.

[104] Nations U. ICTs as a catalyst for sustainable development; 2016. Available from: https://sustainabledevelopment.un.org/index.php?page=view&type=20000&nr=579&menu=2993.

